# Effect of two commercial energy drinks on enamel surface characteristics and orthodontic bracket shear bond strength: an in vitro study

**DOI:** 10.1186/s12903-026-08708-6

**Published:** 2026-06-04

**Authors:** Anan Ashraf, Mohamed Shamel, Caroline Massieh, Ghada Elsaeed, Mahmoud Al Ankily

**Affiliations:** 1 Oral Biology Department, Faculty of Dentistry, The British University in Egypt, Cairo, Egypt; 2 Orthodontic Department, Faculty of Dentistry, The British University in Egypt, Cairo, Egypt

**Keywords:** Energy drinks, Enamel erosion, Surface roughness, Shear bond strength, Orthodontic brackets

## Abstract

**Background:**

Energy drinks are acidic beverages with recognized erosive potential, but their effect on enamel characteristics relevant to orthodontic bonding remains incompletely described. This in vitro study evaluated the effect of continuous 24-hour immersion in two commercial energy drinks, Red Bull and Code Red, on enamel surface morphology, elemental profile, surface roughness, and orthodontic bracket shear bond strength (SBS).

**Methods:**

Thirty sound human lower premolars were randomly allocated to three groups (*n* = 10): distilled water (control), Red Bull, and Code Red. After 24 h of immersion at 37 °C, enamel was assessed using scanning electron microscopy (SEM), energy-dispersive X-ray spectroscopy (EDX), and SEM-derived surface roughness analysis. Stainless-steel premolar brackets were then bonded, and SBS was measured using a universal testing machine.

**Results:**

Under the accelerated laboratory exposure conditions used, both energy drinks produced evident enamel surface alterations compared with the control group. The control group showed the lowest roughness (Ra = 3.04), whereas Red Bull and Code Red increased roughness, with the highest values observed for Code Red (Ra = 24.9). Mean SBS decreased from 24.1 MPa in the control group to 17.7 MPa after Red Bull exposure and 12.7 MPa after Code Red exposure. EDX demonstrated lower calcium values after exposure to both drinks than in the control group.

**Conclusions:**

Within the limitations of this acute in vitro immersion model, both tested energy drinks showed erosive potential and were associated with altered enamel surface characteristics and lower orthodontic bracket SBS compared with distilled water. These findings should be interpreted as laboratory evidence under accelerated exposure conditions rather than as direct evidence of the risk of clinical bracket failure.

## Introduction

Energy drinks (EDs) are functional, non-dairy, non-alcoholic beverages widely consumed worldwide [1]. In recent years, their intake has increased markedly among adolescents and young adults, including groups commonly represented in orthodontic practice [2]. Because these drinks are usually acidic and often contain fermentable sugars, concerns have been raised about their effects on the integrity of dental hard tissues [3, 4].

EDs commonly contain caffeine, organic acids, sugars, B-complex vitamins, and other ingredients such as taurine [5]. From a dental perspective, their low endogenous pH and acid content may lower the oral pH below the critical threshold for enamel dissolution and promote erosive mineral loss [4]. Previous studies have linked exposure to acidic beverages to enamel softening, surface irregularities, and changes in mineral composition, as well as contributing to periodontitis [4, 6].

Previous studies have suggested that acidic EDs, such as Red Bull and Code Red, may contribute to enamel demineralization, particularly with frequent or prolonged exposure [7–9]. However, the extent of this effect depends not only on pH but also on other beverage properties, exposure patterns, and the modifying effects of saliva and the pellicle in vivo [10, 11]. For this reason, laboratory immersion models are best interpreted as controlled erosive challenge models rather than direct replicas of clinical exposure.

The bond strength of orthodontic brackets can be affected by different variables [12]. These include the adhesive system, the enamel surface condition, operator technique, bracket type, and the mechanical stresses encountered intra-orally [12, 13]. Previous in vitro work has shown that enamel surface conditioning methods can materially influence shear bond strength outcomes and should be considered when interpreting bond tests performed after surface challenges [14]. Because bracket retention depends in part on the integrity of the etched enamel surface, erosive alterations before bonding may compromise adhesive performance and increase the risk of bond reduction in vitro.

Although the effect of acidic beverages on enamel has been examined previously, the combined assessment of enamel morphology, elemental profile, roughness, and bracket SBS after exposure to two widely available commercial EDs remains limited. Therefore, this study aimed to evaluate the effect of Red Bull and Code Red on enamel surface characteristics and to assess their subsequent effect on orthodontic bracket SBS under an acute in vitro immersion model.

## Methods

### Specimens preparation

Thirty intact, non-carious human lower premolars extracted for orthodontic treatment were included. Teeth were cleaned, disinfected, and stored in distilled water before use. Written informed consent was obtained from donors, and the protocol was approved by the Research and Ethics Committee of the Faculty of Dentistry, The British University in Egypt (Project No. FD BUE REC 25–058). Donor age and sex were not recorded because all specimens were anonymized, and extracted teeth were collected for laboratory use.

Sample size was calculated using G*Power v3.1 for ANOVA with an expected effect size (f = 0.5), α = 0.05, and power = 0.80, yielding *n* = 10 per group. The teeth were randomly allocated into three groups (*n* = 10) using a computer-generated random number sequence: Group 1, distilled water (control); Group 2, Red Bull; and Group 3, Code Red.

After allocation, each specimen was immersed in its assigned medium at 37 °C for 24 h. This continuous immersion was selected as an accelerated laboratory challenge model, consistent with previous in vitro studies evaluating the effects of acidic beverages on enamel and bracket bond strength [15, 16]. The model was intended to compare erosive potential under standardized conditions and does not simulate salivary buffering, pellicle formation, intermittent intake, or cyclic demineralization/remineralization.

The same teeth were subsequently used for post-exposure surface characterization and SBS testing. Representative SEM images were acquired for qualitative comparison. Quantitative EDX and roughness analyses were generated from the exposed enamel surface, after which brackets were bonded and tested for SBS.

The beverages were freshly opened at the time of testing. Measured beverage acidity values were as follows: Red Bull, pH 3.9 and titratable acidity 7.8 mL of 0.1 N NaOH per 10 mL sample; Code Red, pH 3.7 and titratable acidity 7.9 mL of 0.1 N NaOH per 10 mL sample.

### Scanning Electron Microscopy (SEM), Energy-Dispersive X‐Ray spectroscopy (EDX), and surface roughness analysis

Surface morphology, elemental analysis and surface roughness were performed under the same SEM operating conditions using low-vacuum mode (50 Pa), an accelerating voltage of 20 kV, and a working distance of 13.0 mm. EDX was used to assess the surface elemental composition of enamel, with particular focus on the weight percentages of calcium (Ca) and phosphorus (*P*), as well as the Ca/P ratio [17, 18]. Following experimental exposure, specimens were dehydrated in ascending ethanol concentrations (50%, 70%, 90%, and 100%) for 10 min each, air-dried for 24 h, mounted on aluminum stubs, and examined using variable-pressure SEM in low-vacuum mode (50 Pa) without sputter coating to preserve the native enamel surface. SEM micrographs were captured at ×1300 and ×5000 magnifications, with the ×1300 images used for surface roughness quantification. For each specimen, three non-overlapping ×1300 images were obtained from the mid-buccal enamel surface, avoiding cracks, debris, margins, and preparation artifacts. Images were exported as TIFF files (1280 × 960 pixels) and analyzed using ImageJ (version 1.54d, NIH, USA) with the SurfCharJ plugin. After noise reduction with a median filter, spatial calibration was standardized using the SEM scale bar. A central standardized region of interest was selected from each image, and three line profiles were analyzed per image. The average roughness (Ra, µm) was calculated for each profile, and the mean of nine readings per specimen was recorded as the final Ra value. All images were anonymized before analysis, and the assessor was blinded to group allocation and time point [19].

### Orthodontic bracket bonding

Thirty identical stainless-steel lower premolar brackets (Dentaurum, Ispringen, Germany; base area 10.3 mm²) were bonded to the buccal surfaces after immersion. Enamel was etched with 35% phosphoric acid gel (3 M Unitek, CA, USA) for 15 s, rinsed, and air-dried until a frosty appearance was obtained. Ortho Solo Primer (Ormco, Orange, CA, USA) and Grengloo Adhesive (Ormco, Glendora, CA, USA) were used according to the manufacturer’s instructions. Brackets were seated centrally under a standardized 300 g force for 10 s using a vertically applied calibrated weight. Light curing was performed for 40 s (10 s each from the mesial, distal, occlusal, and gingival aspects) using a 3 M Paradigm DeepCure LED unit (1470 mW/cm²), with the light tip positioned 0–2 mm from the bracket surface [20].

### Shear bond strength (SBS)

The SBS was measured with a universal testing machine (TESTCOM-100, Ibertest, Spain) equipped with a 0-100 kN load cell at a crosshead speed of 1 mm/min. Each tooth was positioned so that the chisel edge contacted the bracket base and applied a force parallel to the tooth surface in an occluso-gingival direction. The debonding force was recorded in Newtons (*N*) and converted to megapascals (MPa) by dividing by the bracket base area (MPa = N/mm²) [21, 22].

### Statistical analysis

Statistical analysis was performed using SPSS version 26.0 (IBM Corp., Chicago, IL, USA). Data distribution was explored using normality testing and inspection of distributional assumptions, and homogeneity of variance was considered before between-group comparisons. Group comparisons were performed using one-way analysis of variance followed by Tukey post hoc testing for pairwise comparisons. Statistical significance was set at *p* < 0.05.

## Results

### Scanning Electron Microscopy (SEM)

**Fig. 1:**
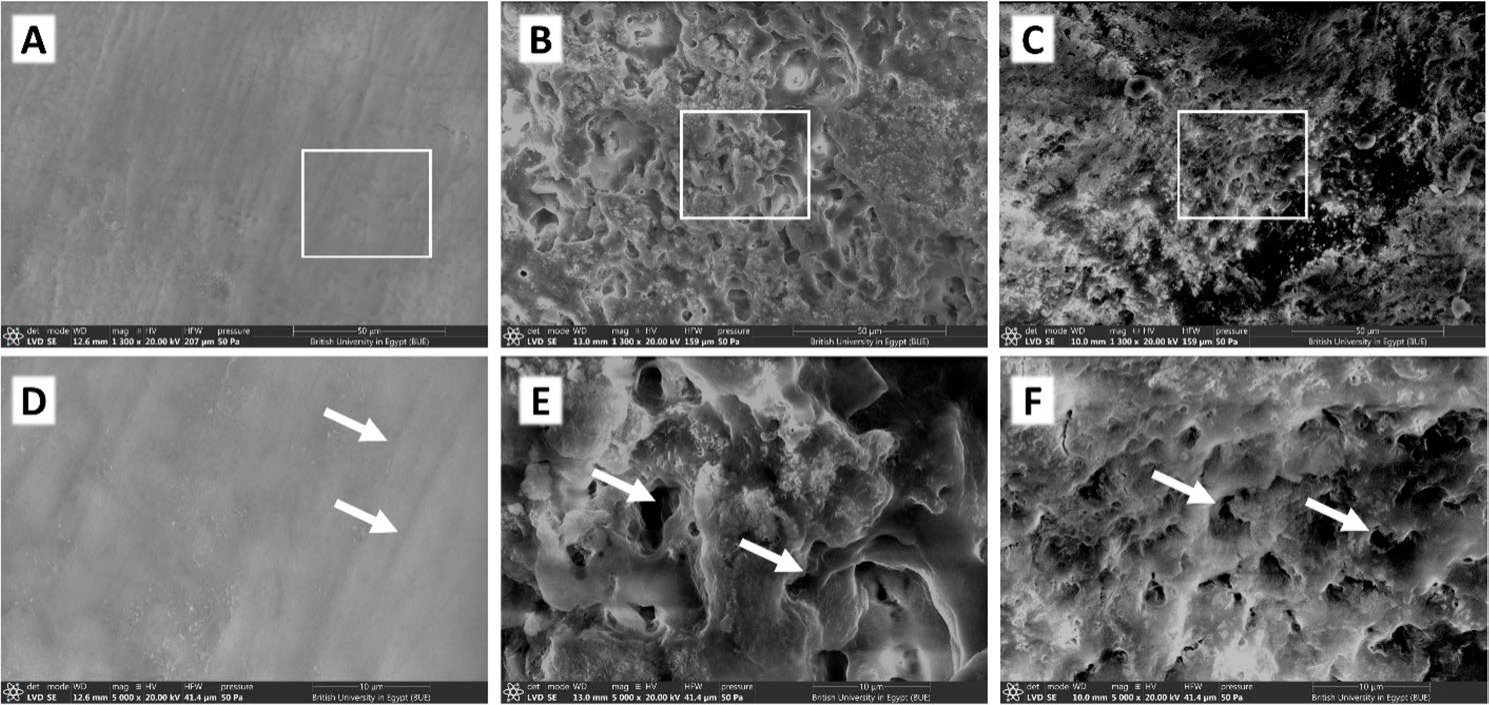
SEM micrographs of enamel surfaces in the control group (**A**, **D**), showing a relatively regular surface topography pattern; the Red Bull group (**B**, **E**), showing depressions and pores; and the Code Red group (**C**, **F**), showing more extensive depressions and amorphous surface changes. **A**-**C**, ×1300 magnification; **D**-**F**, ×5000 magnification

The SEM results showed that the control group presented a regular enamel surface pattern with shallow pores (Fig. 1A, D). Teeth immersed in Red Bull EDs exhibited irregular enamel surfaces with deep depressions and pores (Fig. 1B, E). Teeth immersed in Code Red EDs showed loss of the normal enamel surface structure, with large, deep depressions and amorphous structures (Fig. 1C, F).

### Energy-Dispersive X-ray spectroscopy (EDX)

**Fig. 2:**
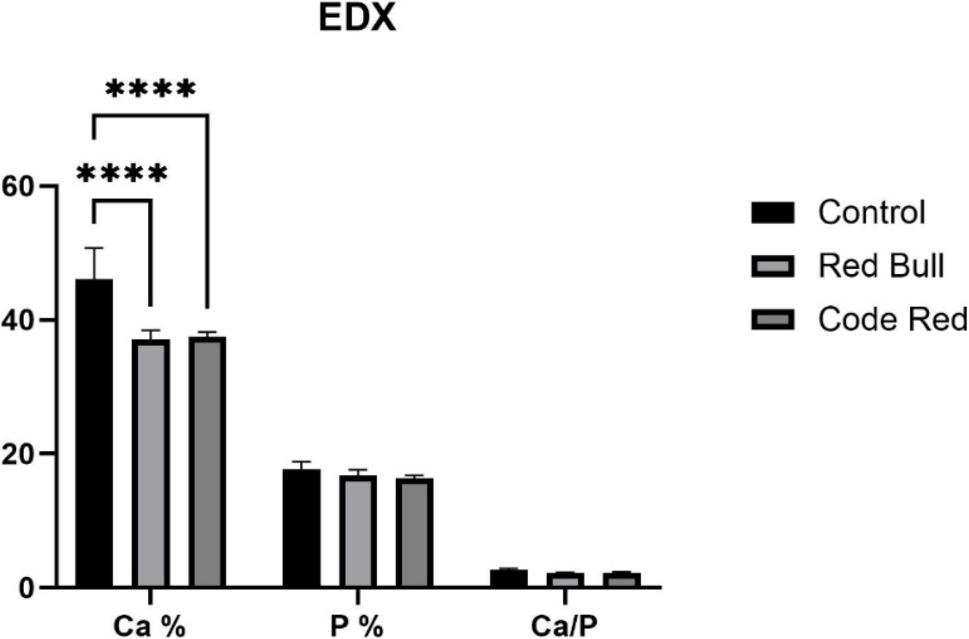
Bar chart showing mean ± SD values of calcium (Ca%), phosphorus (P%), and Ca/P ratio in the control, Red Bull, and Code Red groups. **** indicates significance (*p*<0.0001)

**Fig. 3:**
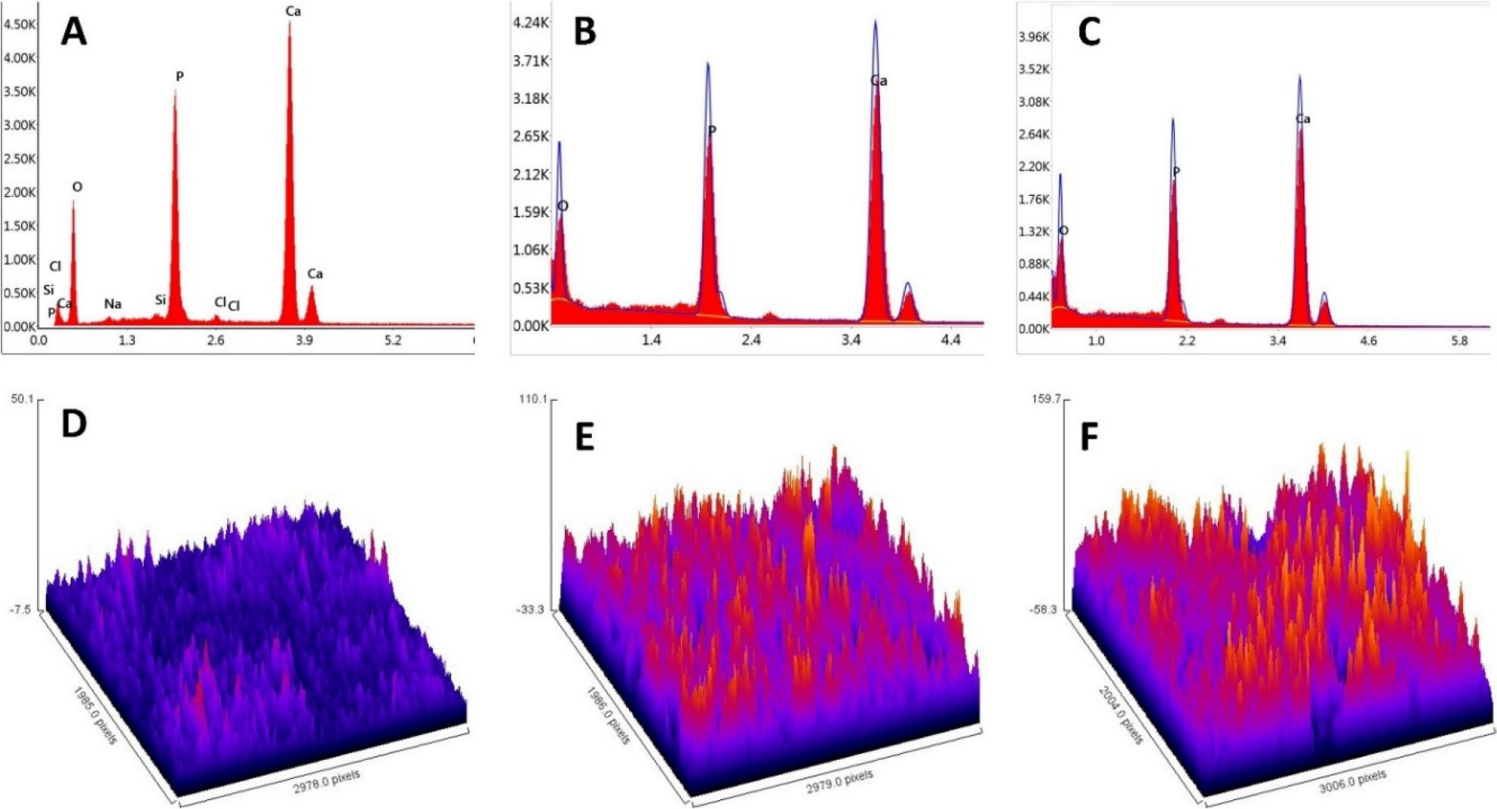
Representative EDX spectra and three-dimensional surface topography of enamel specimens in the study groups. **A**–**C** EDX spectra of enamel specimens from the control group (**A**), Red Bull group (**B**), and Code Red group (**C**). **D**–**F** Three-dimensional surface topography images of enamel specimens from the control group (**D**), Red Bull group (**E**), and Code Red group (**F**), demonstrating differences in surface elevation and depression patterns after exposure to the tested media.

EDX analysis (Figs. 2 and 3, and Tables 1 and 2) showed that exposure to both energy drinks significantly reduced the calcium content of enamel compared with the control group. One-way ANOVA demonstrated a significant overall effect of immersion medium on Ca% (F = 54.21, *p* < 0.0001). The mean Ca% was 46.09 ± 4.65 in the control group, 37.10 ± 1.30 in the Red Bull group, and 37.41 ± 0.77 in the Code Red group. Tukey’s post hoc test showed that Ca% was significantly higher in the control group than in both the Red Bull group (*p* < 0.0001) and the Code Red group (*p* < 0.0001). No significant difference was found between the Red Bull and Code Red groups (*p* = 0.9134).

**Table 1:** EDX analysis of enamel surface elemental composition in the study groups

Parameter	Control (mean ± SD)	Red Bull (mean ± SD)	Code Red (mean ± SD)	*p* value
Calcium (Ca%), wt%	46.09 ± 4.65	37.10 ± 1.30	37.41 ± 0.77	< 0.0001
Phosphorus (*P*%), wt%	17.64 ± 1.20	16.77 ± 0.86	16.40 ± 0.39	0.07
Ca/*P* ratio	2.62 ± 0.24	2.21 ± 0.08	2.28 ± 0.08	0.06

**Table 2:** Tukey’s post hoc multiple comparisons for EDX parameters

Parameter	Comparison	Mean difference	95% CI	Adjusted *p* value
Ca %	Control vs. Red Bull	8.992	7.168 to 10.82	< 0.0001
	Control vs. Code Red	8.682	6.858 to 10.51	< 0.0001
	Red Bull vs. Code Red	-0.310	-2.134 to 1.514	0.9134
*P* %	Control vs. Red Bull	0.870	-0.9545 to 2.694	0.4934
	Control vs. Code Red	1.241	-0.5835 to 3.065	0.2415
	Red Bull vs. Code Red	0.371	-1.453 to 2.195	0.8784
Ca/*P*	Control vs. Red Bull	0.4015	-1.423 to 2.226	0.8591
	Control vs. Code Red	0.3339	-1.491 to 2.158	0.9003
	Red Bull vs. Code Red	-0.0677	-1.892 to 1.757	0.9957

For phosphorus content, one-way ANOVA showed no significant overall effect (*p* = 0.07). The mean P% was 17.64 ± 1.20 in the control group, 16.77 ± 0.86 in the Red Bull group, and 16.40 ± 0.39 in the Code Red group. Although P% was numerically lower in the energy drink groups, the differences were not statistically significant. The mean difference was 0.87 between the control and Red Bull groups (*p* = 0.4934), 1.24 between the control and Code Red groups (*p* = 0.2415), and 0.37 between the Red Bull and Code Red groups (*p* = 0.8784).

Figure 2 Bar chart showing mean ± SD values of calcium (Ca%), phosphorus (P%), and Ca/P ratio in the control, Red Bull, and Code Red groups. **** indicates significance (*p* < 0.0001). Similarly, for the Ca/P ratio, one-way ANOVA showed no significant overall effect, (*p* = 0.06). The mean ratio was 2.62 ± 0.24 in the control group, 2.21 ± 0.08 in the Red Bull group, and 2.28 ± 0.08 in the Code Red group. Although the Ca/P ratio was lower in both energy drink groups than in the control group, these differences were not statistically significant. The mean difference was 0.40 between the control and Red Bull groups, (*p* = 0.8591), 0.33 between the control and Code Red groups, (*p* = 0.9003), and − 0.07 between the Red Bull and Code Red groups, (*p* = 0.9957).

### Surface roughness

**Fig. 4:**
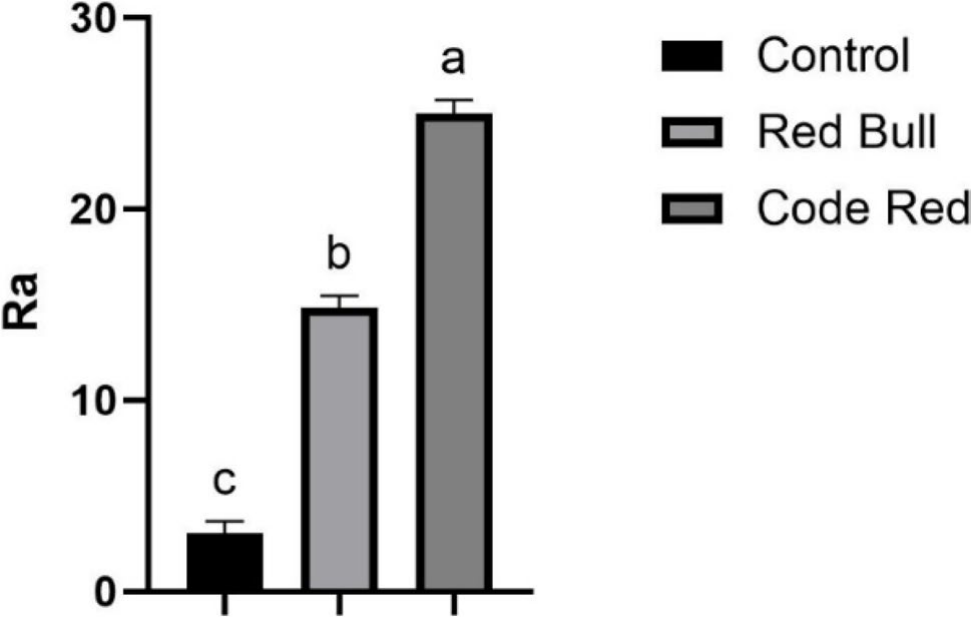
Mean ± standard deviation surface roughness (Ra) values of enamel specimens in the control, Red Bull, and Code Red groups. Different superscript letters indicate statistically significant differences between groups (*p* < 0.05)

Surface roughness analysis showed a highly significant increase in Ra values after exposure to both energy drinks (Fig. 4; Table 3). One-way ANOVA demonstrated a highly significant overall effect of immersion medium on enamel surface roughness (F = 2754, *p* < 0.0001). The mean roughness value was lowest in the control group (3.04 ± 0.63 μm), increased significantly in the Red Bull group (14.83 ± 0.64 μm), and was highest in the Code Red group (24.98 ± 0.71 μm). Tukey’s post hoc test demonstrated significant differences among all groups. Compared with the control group, roughness was significantly higher in the Red Bull group (*p* < 0.0001) and in the Code Red group (*p* < 0.0001). In addition, the Code Red group showed significantly greater roughness than the Red Bull group (*p* < 0.0001). These findings indicate a progressive increase in enamel surface roughness from the control group to Red Bull and then to Code Red.

**Table 3:** Surface roughness (Ra, µm) of enamel specimens and Tukey’s post hoc pairwise comparisons

Group / Comparison	Mean ± SD	Mean difference	95% CI	Adjusted *p* value
Control	3.04 ± 0.63	—	—	—
Red Bull	14.83 ± 0.64	—	—	—
Code Red	24.98 ± 0.71	—	—	—
Control vs. Red Bull	—	-11.79	-12.52 to -11.06	< 0.0001
Control vs. Code Red	—	-21.94	-22.68 to -21.21	< 0.0001
Red Bull vs. Code Red	—	-10.15	-10.89 to -9.42	< 0.0001

### Shear bond strength (SBS) 

**Fig. 5:**
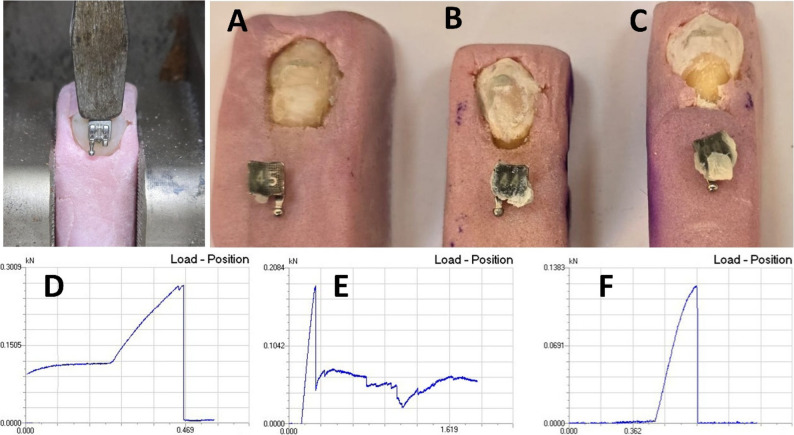
Representative debonding patterns and load–position curves after SBS testing. **A**–**C** Stereomicroscopic images of bracket bases after debonding in the control (**A**), Red Bull (**B**), and Code Red (**C**) groups. The control group showed mostly clean bracket bases, the Red Bull group showed more frequent adhesive remnants, and some Code Red specimens showed enamel fragments attached to the bracket base. D–F Representative load–position curves for the control (**D**), Red Bull (**E**), and Code Red (**F**) groups, showing the highest peak load in the control group, an intermediate peak load in the Red Bull group, and the lowest peak load in the Code Red group

After debonding, different failure appearances were observed across groups (Fig. 5). The control group generally showed cleaner bracket bases, whereas the Red Bull group showed more frequent adhesive remnants and some Code Red specimens showed residual enamel fragments. Because failure mode was not scored using a standardized adhesive remnant index, these observations are presented descriptively only.

**Fig. 6:**
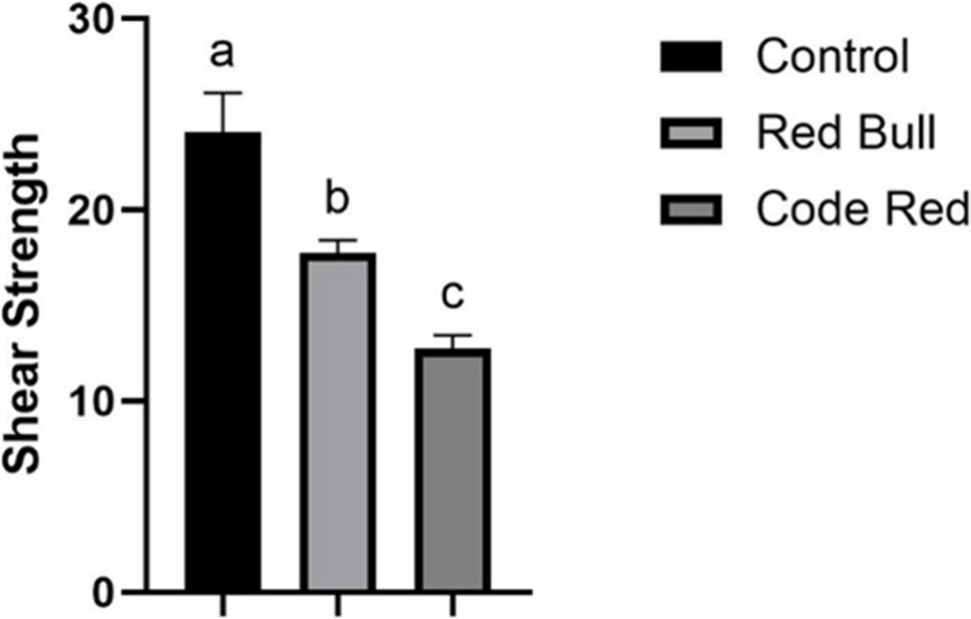
Mean ± SD shear bond strength (MPa) values in the control, Red Bull, and Code Red groups. Different superscript letters denote statistically significant differences between groups (*p* < 0.05)

Shear bond strength analysis (Fig. 6; Table 4) showed significant differences among all groups. One-way ANOVA demonstrated a highly significant overall effect of immersion medium on SBS (F = 154.8, *p* < 0.0001). The mean SBS was highest in the control group (24.1 ± 2.05 MPa), followed by the Red Bull group (17.7 ± 0.64 MPa), and lowest in the Code Red group (12.7 ± 0.70 MPa). Tukey’s post hoc test demonstrated that SBS was significantly lower in the Red Bull group than in the control group (*p* < 0.0001), and significantly lower in the Code Red group than in the control group (*p* < 0.0001). In addition, the Code Red group showed significantly lower SBS than the Red Bull group (*p* = 0.0001).

**Table 4:** Shear bond strength (MPa) of enamel specimens in the study groups and Tukey’s post hoc pairwise comparisons

Group / Comparison	Mean ± SD	Mean difference	95% CI	Adjusted *p* value
Control	24.1 ± 2.05	—	—	—
Red Bull	17.7 ± 0.64	—	—	—
Code Red	12.7 ± 0.70	—	—	—
Control vs. Red Bull	—	6.29	4.09 to 8.49	< 0.0001
Control vs. Code Red	—	11.31	9.11 to 13.51	< 0.0001
Red Bull vs. Code Red	—	5.02	2.82 to 7.22	0.0001

## Discussion

The present study evaluated the effect of two commercial energy drinks, Red Bull and Code Red, on enamel surface characteristics and orthodontic bracket SBS using a continuous 24-hour in vitro immersion model. Under these accelerated laboratory exposure conditions, both beverages were associated with enamel surface alteration, lower surface calcium values, higher roughness, and lower bracket SBS than distilled water.

SEM examination showed progressive surface degradation in the energy-drink groups, with irregularities, depressions, and porosity, compared with the more regular surface of the control specimens. These findings are consistent with previous studies reporting erosive alterations in enamel after exposure to acidic beverages [23–25]. However, the present data should be interpreted as evidence of erosive potential within an acute laboratory stress model rather than as direct evidence of what occurs clinically after ordinary beverage consumption.

The EDX findings showed that exposure to both energy drinks significantly reduced enamel calcium content compared with the control group, whereas the reductions in phosphorus content and Ca/P ratio were not statistically significant. Specifically, both Red Bull and Code Red produced a marked decrease in Ca%, with no significant difference between the two beverages. This suggests that both drinks exerted a comparable demineralizing effect on the calcium component of the enamel surface under the conditions tested. In contrast, although P% and Ca/P ratio were numerically lower in the energy drink groups, the lack of statistical significance indicates that these changes were less pronounced and may have been limited to superficial mineral alterations. These findings support the view that early erosive changes induced by acidic beverages may preferentially affect calcium loss from the enamel surface before producing broader detectable changes in overall mineral balance. They are also consistent with previous studies reporting that acidic drinks can reduce enamel mineral content because of their low pH and erosive potential [4, 23].

The surface roughness findings further confirmed the erosive effect of both energy drinks on enamel. Mean Ra values increased significantly after exposure to Red Bull and Code Red compared with the control group, with Code Red showing the highest roughness and Red Bull exhibiting an intermediate effect. Moreover, all pairwise comparisons were statistically significant, indicating that the two beverages differed not only from the control condition but also from each other in the magnitude of surface alteration produced. These findings suggest that exposure to acidic energy drinks causes substantial physical deterioration of the enamel surface under the conditions tested, and that Code Red exerted a greater roughening effect than Red Bull. This pattern is consistent with the SEM observations and supports the interpretation that the tested beverages increased enamel surface irregularity and surface loss [26–28].

Taken together, the surface morphology, EDX, and roughness findings indicate that the effects of the tested drinks were not only visual, but also compositional and topographical. In the present study, both beverages were acidic, with measured pH values of 3.9 for Red Bull and 3.7 for Code Red. Their titratable acidity values were also very similar (7.8 and 7.9 mL of 0.1 N NaOH per 10 mL sample, respectively), indicating that both drinks had substantial acid-neutralizing demand. These measured values support the interpretation that both beverages had erosive potential under the experimental conditions used. However, because sugar content, buffering characteristics, and the full compositional profile were not independently analyzed in this study, mechanistic comparisons between Code Red and Red Bull should still be interpreted cautiously.

The SBS findings showed that exposure to both energy drinks significantly reduced bracket bond strength, with the lowest values observed in the Code Red group and intermediate values in the Red Bull group. Moreover, all pairwise comparisons were statistically significant, indicating that both beverages adversely affected bonding performance and that the reduction was more pronounced after Code Red exposure than after Red Bull exposure. When interpreted in relation to commonly cited clinically acceptable SBS ranges (minimum 6–8 MPa) for orthodontic bonding, the Red Bull group remained within a range that may still be considered clinically acceptable, whereas the Code Red group approached the lower end of that range [29–31]. Therefore, although both beverages reduced bond strength under the accelerated in vitro conditions used in this study, the magnitude of reduction appears more clinically concerning for Code Red than for Red Bull.

The present study has several limitations. First, it used a continuous 24-hour immersion protocol that represents an acute, accelerated challenge rather than a direct simulation of real-life drinking behavior. Salivary buffering, pellicle formation, oral clearance, thermal changes, and cyclic demineralization/remineralization were not reproduced [32]. Second, although pH and titratable acidity were measured for the tested beverages, other potentially relevant physicochemical variables, such as sugar concentration, calcium/phosphate content, and buffering behavior, were not independently analyzed; therefore, mechanistic interpretation should be cautious. Third, failure mode analysis was descriptive only, because no standardized adhesive remnant index was used. Despite these limitations, the study provides useful comparative laboratory evidence that both tested commercial energy drinks can adversely affect enamel-related outcomes relevant to orthodontic bonding under controlled experimental conditions. Clinically, these results support prudent dietary counseling for patients with orthodontic appliances and consideration of preventive strategies such as fluoride exposure and reduction of frequent acidic-drink intake [33].

## Conclusions

Within the limitations of this acute in vitro immersion model, both tested commercial energy drinks showed erosive potential and were associated with altered enamel surface morphology, lower surface calcium values, increased surface roughness, and reduced orthodontic bracket SBS compared with distilled water. These results should be interpreted as laboratory findings under accelerated exposure conditions and not as direct evidence of clinical bracket failure risk.

## Data Availability

The data supporting the findings of the present study are available upon reasonable request.

## Abbreviations

Energy drink: ED
EDX: Energy-dispersive X-ray spectroscopy
SEM: Scanning electron microscopy
SBS: Shear bond strength
